# Brow Fat Transposition for Reconstruction of Hollowing After Dermoid Cyst Excision on the Outer Upper Eyelid in a Young Woman: A Case Report

**DOI:** 10.7759/cureus.100571

**Published:** 2026-01-01

**Authors:** Shiho Komai, Yoshiyuki Kitaguchi, Rikako Iwasaki, Takeshi Morimoto, Shimpei Komoto, Hiroshi Shimojyo, Takahiro Fujino, Kohji Nishida

**Affiliations:** 1 Department of Ophthalmology, Hyogo Prefectural Nishinomiya Hospital, Nishinomiya, JPN; 2 Department of Ophthalmology, The University of Osaka Graduate School of Medicine, Suita, JPN; 3 Department of Advanced Visual Neuroscience, The University of Osaka Graduate School of Medicine, Suita, JPN

**Keywords:** augmentation, brow fat, dermoid cyst, hollowing, roof

## Abstract

Periocular hollowing following tumor excision presents unique reconstructive challenges, particularly in young patients, as the literature on post-excisional reconstruction remains limited compared with that on age-related volume loss. We report the case of a 13-year-old female who presented with a dermoid cyst in the right upper eyelid causing mechanical ptosis. Following complete excision, a significant hollow developed at the surgical site. A single-stage reconstruction was performed using a pedicled brow fat (retro-orbicularis oculi fat) flap transposition to address the volume deficit. At the 18-month follow-up, excellent contour restoration and symmetry were achieved without complications. This case highlights that brow fat transposition offers a reliable, single-stage reconstructive option for upper eyelid hollowing following tumor excision, providing stable autologous volume augmentation with favorable aesthetic outcomes in young patients.

## Introduction

Periocular hollowing, characterized by a recessed appearance around the eyes, represents both an aesthetic and functional concern [1]. While primarily associated with age-related fat atrophy and skin laxity, hollowing also occurs following trauma or tissue loss after eyelid tumor excision. In cases of long-standing benign tumors such as dermoid cysts, the mass volume often masks underlying soft tissue atrophy caused by chronic compression. Consequently, significant hollowing may only become apparent after the tumor is excised. Various surgical and nonsurgical techniques address age-related upper eyelid hollowing, including orbital fat repositioning [2], brow fat pad transfer [3], orbicularis flaps [2], autologous fat grafting [4,5], and periorbital filler injections [6,7]. These methods restore periorbital volume and contour. However, the literature regarding reconstructive techniques for post-tumor excision hollowing, particularly in younger patients, remains limited.

We present a successful reconstruction of lateral upper eyelid hollowing following dermoid cyst excision in a young woman using single-stage brow fat transposition.

## Case presentation

**Figure 1:**
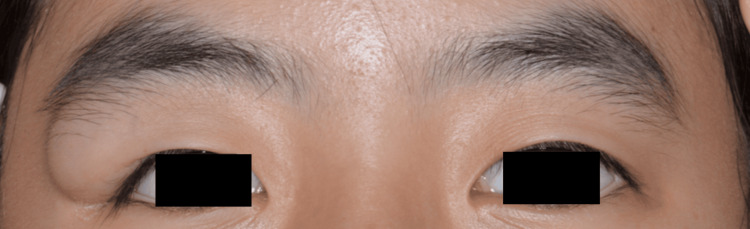
Preoperative frontal view photograph showing a visible mass in the right upper eyelid

A 13-year-old female presented with a progressively enlarging right upper eyelid mass that had been present since infancy. She had no history of trauma, prior ophthalmic surgery, or systemic disease. Corrected distance visual acuity was 20/20 bilaterally, without diplopia. Examination revealed a soft, nontender, mobile mass measuring approximately 20 × 10 mm in the lateral right upper eyelid, causing mild mechanical ptosis (Figure 1). No inflammatory signs were present. Computed tomography demonstrated a well-defined, low-density, extraconal preseptal mass without bony erosion, consistent with a dermoid cyst. No preoperative hollowing was evident on imaging because the mass volume occupied the subcutaneous space.

**Figure 2:**
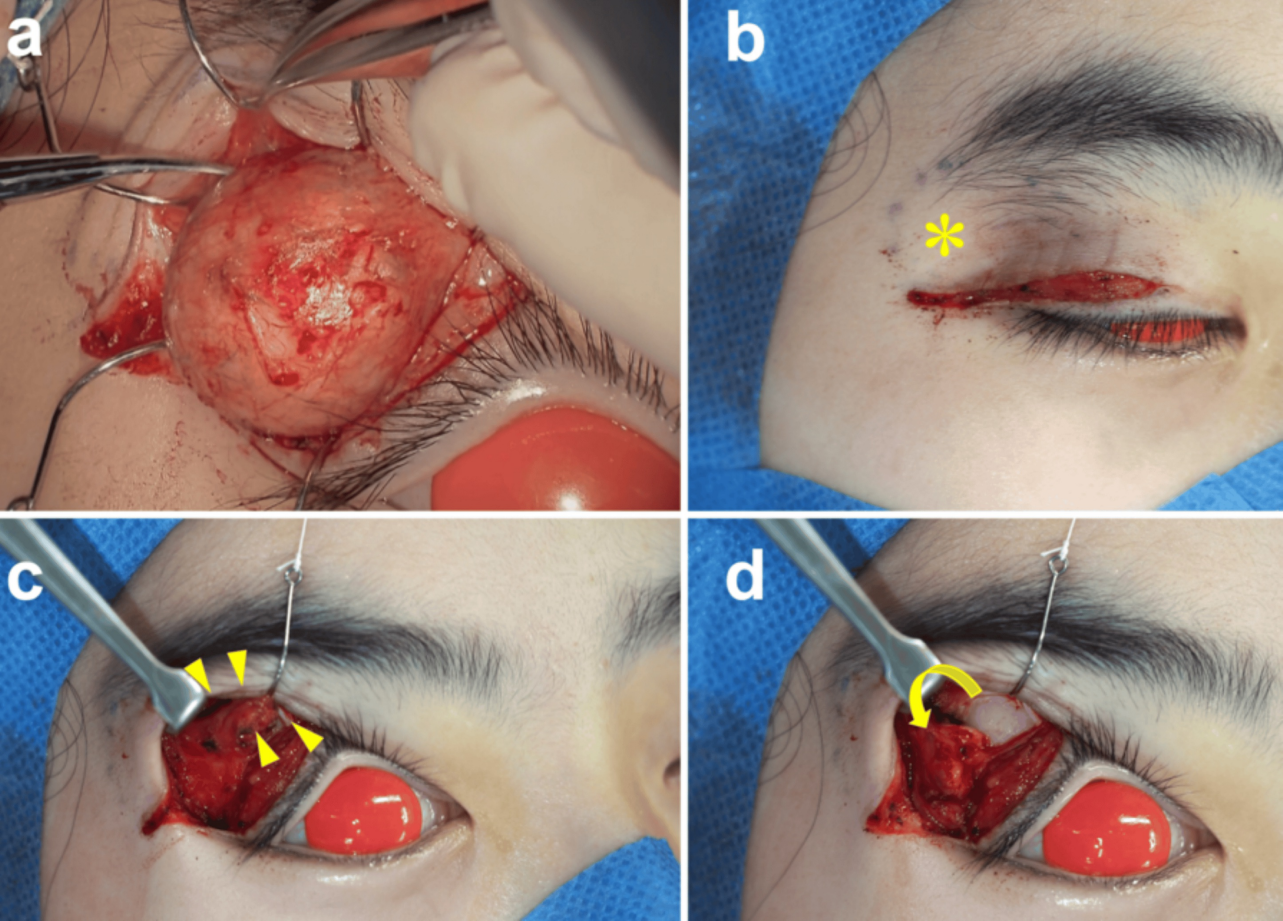
Intraoperative photographs demonstrating the surgical technique (a) Exposed dermoid cyst after skin incision along the eyelid crease. (b) Post-excisional hollowing (asterisk) following complete tumor removal. (c) Creation of an inferolaterally based pedicled flap comprising brow fat and underlying periosteum (arrowheads). (d) Volume augmentation achieved by inferior rotation and transposition of the flap into the defect.

Under general anesthesia, a horizontal incision was made along the natural eyelid crease. The mass, located anterior to the orbital septum and adherent to the tarsal plate, was meticulously dissected and excised en bloc with an intact capsule to prevent content spillage (Figure 2). After excision, significant depression was noted at the surgical site due to both volume deficit and attenuation of chronically compressed pretarsal tissues. For reconstruction, dissection was extended superiorly in the suborbicularis plane approximately 10 mm above the superior orbital rim, exposing the brow fat pad. An inferolaterally based pedicled flap comprising brow fat and underlying periosteum was designed, elevated from the frontal bone, rotated inferiorly, and transposed into the defect for lateral upper eyelid augmentation. After redraping the skin, a conservative amount of redundant skin was trimmed to address skin redundancy. Layered closure of the orbicularis oculi and skin was performed.

**Figure 3:**
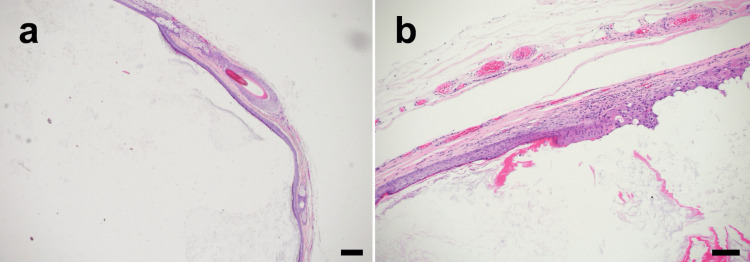
Histopathological findings of the excised dermoid cyst showing a stratified squamous epithelial lining with loose connective tissue in the cyst cavity Hematoxylin and eosin staining. Scale bars: (a) 200 μm, (b) 100 μm.

**Figure 4:**
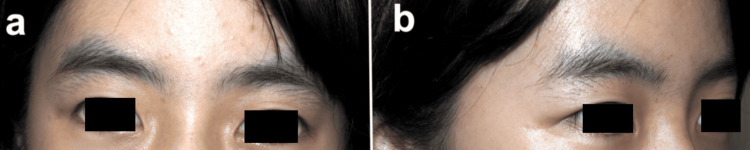
Postoperative photographs at the 18-month follow-up showing (a) frontal and (b) oblique views Note the symmetric upper eyelid contour and complete restoration of lateral volume.

Histopathological examination confirmed a dermoid cyst (Figure 3). The 18-month follow-up revealed a smooth, symmetric upper eyelid contour with effective correction of the depression and an inconspicuous surgical scar within the eyelid crease (Figure 4).

## Discussion

This case demonstrates successful single-stage reconstruction of iatrogenic upper eyelid hollowing using a pedicled brow fat flap following dermoid cyst excision, effectively addressing the post-excisional volume deficit with a stable cosmetic outcome in a young patient. While skin remodeling can reduce superficial redundancy over time, it is insufficient to correct deep volume deficits caused by chronic tissue atrophy [1]. Therefore, immediate autologous volume augmentation was indicated to restore the periorbital contour, as volume loss requires structural replacement rather than simple skin tightening [4,5].

Alternative upper eyelid augmentation methods present various limitations. The long-term outcomes of autologous fat grafting are unpredictable, primarily because the resorption rate of the grafted fat is variable, which may necessitate secondary procedures [4,5]. Preaponeurotic orbital fat repositioning is unsuitable for patients lacking excess orbital fat and with defects confined to the preseptal space [2]. Nonsurgical options such as hyaluronic acid fillers are inappropriate for young patients requiring permanent structural solutions because of their temporary effect and potential complications [6,7].

The retro-orbicularis oculi fat (ROOF) flap provided an optimal solution. This well-vascularized fat pad, located deep to the orbicularis oculi muscle, demonstrates reliability as a local flap and has been traditionally used in aesthetic surgery [1,3,8]. Primary advantages include the use of adjacent autologous tissue, eliminating separate donor sites and minimizing surgical morbidity. As a pedicled flap, it ensures a stable blood supply with predictable, permanent volume augmentation without the resorption risk associated with free fat grafting [4,5]. The flap’s versatility allows precise tailoring to specific anatomical defects, providing customized structural support.

This technique has inherent limitations that warrant consideration. The volume of the ROOF flap is anatomically variable among individuals, with some patients having insufficient tissue for reconstruction of substantial defects. Technical challenges include precise dissection to preserve the vascular supply while avoiding injury to the supraorbital nerve, which may result in transient numbness, as reported in up to 20% of cases following ROOF resection procedures [9]. Long-term outcomes beyond our 18-month follow-up period require further investigation to fully evaluate volume stability and aesthetic durability.

## Conclusions

Pedicled brow fat pad transposition successfully provides single-stage reconstruction for upper eyelid hollowing following tumor excision. By utilizing adjacent vascularized tissue, this technique minimizes donor site morbidity and avoids the unpredictability of free fat grafting or synthetic fillers. It offers a stable, cosmetically superior solution, particularly valuable for young patients requiring long-term autologous volume augmentation. However, further studies involving larger case series and longer follow-up periods are required to validate the broader applicability and long-term efficacy of this technique.

## References

[1] Goldberg RA (2005). The three periorbital hollows: a paradigm for periorbital rejuvenation. Plast Reconstr Surg.

[2] Li X, Xia L, Ma L (2023). Sunken upper eyelid deformity correction by orbital fat pad repositioning and orbicularis oculi muscle folding in blepharoplasty. J Craniofac Surg.

[3] Qu L, Liang Z, Wang J, Zhang J, Yu Z, Song B (2024). Combined blepharoplasty and brow fat pad transfer for the correction of dermatochalasis and sunken upper eyelids. Plast Reconstr Surg.

[4] Ramil ME (2017). Fat grafting in hollow upper eyelids and volumetric upper blepharoplasty. Plast Reconstr Surg.

[5] Yang F, Ji Z, Peng L (2021). Efficacy, safety and complications of autologous fat grafting to the eyelids and periorbital area: a systematic review and meta-analysis. PLoS ONE.

[6] Tan P, Kwong TQ, Malhotra R (2018). Non-aesthetic indications for periocular hyaluronic acid filler treatment: a review. Br J Ophthalmol.

[7] Berros P (2010). Periorbital contour abnormalities: hollow eye ring management with hyalurostructure. Orbit.

[8] Maniglia JJ, Maniglia RF, Jorge dos Santos MC, Robert F, Maniglia FF, Maniglia SF (2006). Surgical treatment of the sunken upper eyelid. Arch Facial Plast Surg.

[9] May JW Jr, Fearon J, Zingarelli P (1990). Retro-orbicularis oculus fat (ROOF) resection in aesthetic blepharoplasty: a 6-year study in 63 patients. Plast Reconstr Surg.

